# Targeting *MET* Amplification: Opportunities and Obstacles in Therapeutic Approaches

**DOI:** 10.3390/cancers15184552

**Published:** 2023-09-14

**Authors:** Yuichi Kumaki, Goshi Oda, Sadakatsu Ikeda

**Affiliations:** 1 Department of Specialized Surgery, Tokyo Medical and Dental University, Tokyo 113-8519, Japan; odasrg2@tmd.ac.jp; 2 Center for Innovative Cancer Treatment, Tokyo Medical and Dental University, Tokyo 113-8519, Japan; 3 Moores Cancer Center, University of California San Diego, La Jolla, CA 92037, USA

**Keywords:** *MET* amplification, polysomy, aneuploidy, acquired resistance, MET inhibitor

## Abstract

**Simple Summary:**

The *MET* gene is crucial for cell growth and has shown promise as a cancer treatment target. However, distinguishing between focal amplification and polysomy, different types of gene multiplication, is challenging. Accurate differentiation requires techniques such as in situ hybridization (ISH) or next generation sequencing (NGS). As the effectiveness of MET inhibitors can vary, careful patient selection and defining the perfect amplification threshold are critical. Future studies should focus on determining optimal therapy combinations and innovating new treatments targeting *MET* amplification.

**Abstract:**

The *MET* gene plays a vital role in cellular proliferation, earning it recognition as a principal oncogene. Therapies that target *MET* amplification have demonstrated promising results both in preclinical models and in specific clinical cases. A significant obstacle to these therapies is the ability to distinguish between focal amplification and polysomy, a task for which simple *MET* copy number measurement proves insufficient. To effectively differentiate between the two, it is crucial to utilize comparative measures, including in situ hybridization (ISH) with the centromere or next generation sequencing (NGS) with adjacent genes. Despite the promising potential of *MET* amplification treatment, the judicious selection of patients is paramount to maximize therapeutic efficacy. The effectiveness of MET inhibitors can fluctuate depending on the extent of *MET* amplification. Future research must seek to establish the ideal threshold value for *MET* amplification, identify the most efficacious combination therapies, and innovate new targeted treatments for patients exhibiting *MET* amplification.

## 1. Introduction

The *MET* gene, also referred to as c-MET, encodes a receptor tyrosine kinase (RTK) known as MET. This gene has garnered significant attention in the oncology field due to its pivotal role in tumorigenesis and metastasis [1]. The aberrant activation of MET signaling has been implicated in the development and progression of several malignancies, including non-small cell lung cancer (NSCLC) [2], gastric cancer [3,4], colorectal cancer [5], papillary renal cell carcinoma (PRCC) [6], hepatocellular carcinoma [7], and breast cancer [8,9].

Multiple genetic alterations within *MET* have been identified as oncogenic drivers in cancer. One such alteration is exon 14 skipping, which results in an aberrant MET protein lacking a critical regulatory domain [10]. The event leads to constitutive activation of the MET signaling pathway and contributes to tumorigenesis, particularly in NSCLC [11]. Notably, studies have revealed that approximately 3% of NSCLC patients exhibit *MET* exon 14 skipping, highlighting its clinical relevance [10].

In addition to exon 14 skipping, gene amplification and single nucleotide variants have been implicated in MET-driven oncogenesis. *MET* amplification is characterized by an increased copy number of the *MET* gene, leading to elevated MET protein levels and hyperactivation of downstream signaling cascades, including the RAS-ERK/MAPK, PI3K-AKT-mTOR, or PLCgamma-PKC pathways [12,13,14,15]. This phenomenon has been observed in various cancer types and holds promise as a therapeutic target [16,17].

The identification of specific *MET* alterations has paved the way for targeted therapies in cancer treatment. For instance, in the case of NSCLC patients with *MET* exon 14 skipping, the development of MET tyrosine kinase inhibitors (TKIs), such as capmatinib and tepotinib, has shown remarkable efficacy in clinical trials [18,19,20]. These TKIs selectively inhibit the activated MET kinase, thereby suppressing aberrant MET signaling and impeding tumor growth.

Furthermore, *MET* amplification has emerged as an intriguing therapeutic target. Targeted therapies designed to counteract *MET* amplification have shown promise in preclinical studies and a subset of clinical studies [18,21]. However, challenges remain, particularly regarding the development of effective inhibitors that can overcome resistance mechanisms associated with *MET* amplification [22,23,24,25,26].

In light of the significance of *MET* alterations in cancer, this review aims to comprehensively summarize the measurement, frequency, and therapeutic implications of *MET* amplification. We seek to elucidate the underlying mechanisms driving *MET* amplification and explore its potential as a therapeutic target. Additionally, we will discuss the challenges and prospects associated with targeting *MET* amplification, including the emergence of resistance mechanisms.

## 2. Gene Amplification and Protein Overexpression

Gene amplification is a prevalent genetic alteration observed in cancer [27,28,29]. It usually causes protein overexpression by enhancing levels of the products encoded by the amplified gene [27]. Although gene amplification and protein overexpression are distinct phenomena, they are often associated with each other [27,28]. In general, protein overexpression of RTKs can have oncogenic effects by increasing local receptor concentration, leading to auto-dimerization of receptors, and subsequent hyperactivation of downstream signaling pathways [27]. For instance, amplification of the *HER2* (*ERBB2*) gene on chromosome 17 and the overexpression of HER2 protein play a crucial role in the pathogenesis of HER2-positive breast cancer [30]. Fluorescent in situ hybridization (FISH) is a method that detects *ERRB2* gene amplification, while HER2 immunochemistry (IHC) identifies overexpression of HER2 protein. These two assays are commonly used and exhibit a strong correlation, serving as biomarkers for anti-HER2 therapy [31,32]. In the clinical setting of breast cancer, HER2 status determination often involves measuring HER2 protein expression using IHC initially, followed by FISH when the IHC result is equivocal (2+). Targeted therapies specially designed for HER2-positive breast cancer have demonstrated remarkable success, making them the most effective treatment in the realm of personalized medicine [33].

However, the situation regarding MET differs significantly from HER2. Although the method of measurement and threshold for *MET* amplification is controversial, *MET* amplification has been reported in a number of cancer types. It occurs 1% to 6% in NSCLC patients [34,35,36,37], 1% to 10% in gastric cancers [3,4,38,39], 1% to 4% in colorectal cancers [40,41], 3% to 13% in PRCCs [42], and 8% in breast cancers [43].

It has been observed that MET protein expression assessed by IHC does not always align with *MET* amplification [44,45,46]. Therefore, it is crucial to recognize that the patient populations identified by *MET* amplification techniques such as FISH and next generation sequencing (NGS) may differ substantially from those identified using IHC for treatment selection. The underlying reasons for the discordance between MET protein expression and gene amplification are not yet fully understood, but intra-tumor heterogeneity has been suggested as a contributing factor [46,47]. Previous studies investigating targeted therapies for MET based on IHC measurements have yielded inconsistent results [48,49,50]. Consequently, IHC may not be the appropriate approach to identifying the population that would benefit from MET-targeted therapy.

## 3. Focal Gene Amplification and Polysomy

Gene copy number gain in cancer can manifest as either focal gene amplification or polysomy (or aneuploidy of chromosome) [51]. Focal amplification refers to the specific gain of gene copies in the specific gene, while polysomy involves an overall increase in chromosome copy number [51]. In the case of HER2, FISH is employed to measure gene copy number (GCN) and the ratio of *ERRB2* to centromere enumerator probe (CEP)17. However, it is crucial to consider the presence of polysomy when interpreting FISH results [52]. Polysomy has been identified as the primary cause of equivocal HER2 FISH results, and its association with the efficacy or prognosis of trastuzumab treatment remains unclear [53,54]. Consequently, the selection of appropriate candidates for anti-HER2 therapy requires careful consideration [55].

**Figure 1:**
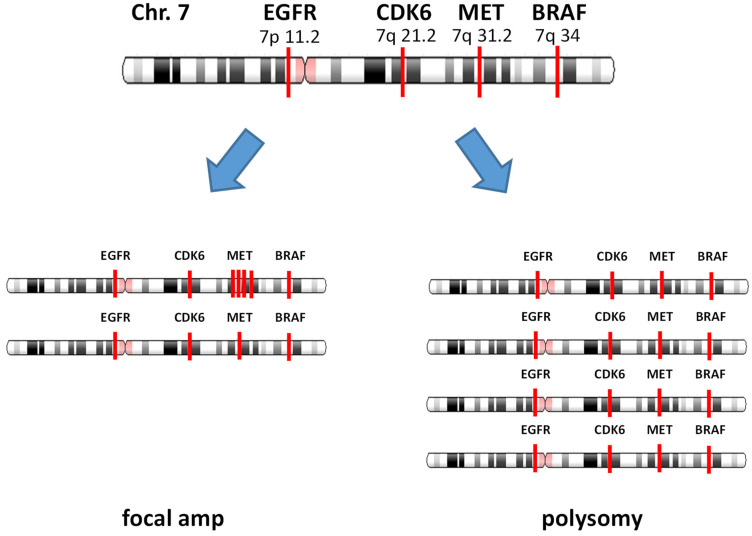
Schematic representation of focal gene amplification and polysomy involving the *MET* gene and chromosome 7 copy number alterations.

Similarly, in the context of MET, focal amplification of the *MET* gene entails a specific gain of gene copies, while polysomy involves an increase in chromosome 7 copy number, often accompanied by co-amplification of adjacent genes such as *CDK6* and *BRAF* [56] (Figure 1). It has been observed that polysomy does not exhibit a favorable response to treatment with MET inhibitors alone [57]. Currently, there is a lack of standardized methods, including the choice of diagnostic tools and the establishment of thresholds, to effectively define focal *MET* amplification as a therapeutic target [56,58].

## 4. *MET* Amplification Detection by FISH

Various methods, such as FISH and NGS, have been employed to detect *MET* amplification [33]. Among these methods, FISH has been regarded as the gold standard for measuring *MET* amplification [59,60,61]. In previous studies, *MET* amplification was defined based on the *MET*/CEP7 ratio, using FISH analysis, and it was found to occur in approximately 5% of patients with NSCLC or gastric adenocarcinoma, with a cut-off value of *MET*/CEP > 2.2 [16]. The correlation between FISH and IHC is under investigation.

However, a phase I study reported that patients with *MET* amplification, defined by a cut-off of *MET*/CEP ≥ 2.0, did not exhibit a favorable response to therapy with a MET inhibitor [62]. This raises concerns regarding the accurate selection of the target population for *MET* amplification-directed treatment. Further investigations utilizing FISH measurements have revealed that only cases with high *MET*/CEP ratios, such as *MET*/CEP > 5, exhibit a favorable response to MET inhibitors [63,64,65]. Guo et al. conducted a comprehensive analysis of the relationship between the definition of *MET* amplification by FISH and the response rate to targeted therapy in previous clinical trials. They consistently observed the most favorable outcomes in NSCLCs characterized by high-level *MET* amplification [57].

It is worth noting that the efficacy of MET inhibitors alone may be diminished when *MET* amplification coexists with other driver mutations. In such cases, combination therapies may be necessary to achieve optimal treatment outcomes.

## 5. *MET* Amplification Detection by NGS

*MET* amplification detection using NGS has gained popularity in clinical settings, offering a comprehensive analysis of genomic alterations. However, its efficacy as a tool for identifying optimal biomarkers to guide MET therapy remains a topic of debate. Several studies have indicated that NGS may not be as effective as FISH in predicting response to MET inhibitors [66,67]. For instance, a comparative analysis between NGS-based gene copy number (GCN) assessment and FISH revealed that NGS alone is not sufficient for predicting MET inhibitor response [68]. One potential limitation of NGS is its inability to accurately distinguish between polysomy and focal amplification, which may impact the identification of suitable therapeutic targets. This might stem from inaccurate interpretation of copy number gain result. Without recognizing the potential polysomy, the inaccurate interpretation of copy number gain might occur. Strategies to address this challenge are still under investigation, and a recent study proposed a method to differentiate polysomy from focal *MET* amplification by co-examining the amplification of genes on chromosome 7, such as *BRAF* and *CDK6* [56].

Our group analyzed the data from 1025 patients with advanced solid tumors using a non-invasive cfDNA NGS panel known as Guardant360 [56]. An algorithm to define focal amplification was developed. Focal amplification was defined as *MET* amplification without polysomy or an increase in the chromosome copy number itself. The Guardant360 test examined four genes located on chromosome 7: *EGFR* in 7p11.2, *CDK6* in 7q21.2, *MET* in 7q31.2, and *BRAF* in 7q34. The *MET* gene was classified as focally amplified if there were no co-amplification of adjacent genes, such as *CDK6* or *BRAF*. Conversely, *MET* non-focal amplification was defined as a *MET* copy number increase associated with polysomy, in which *MET* copy number increased together with either *CDK6* and/or *BRAF*.

Several examples were given to illustrate this: a sample with only *MET* amplification would be classified as focal. If a sample had co-amplification of *MET* and *EGFR* without *CDK6* amplification, it would be categorized as focal amplification, given that polysomy could not occur without increasing the copy numbers of all three genes together. The algorithm to describe focal amplification was defined as follows:

(a) *MET* copy number ≥ 2.2.
(b) *MET* is amplified without co-amplification of *CDK6* and *BRAF*. Co-amplification status was defined as “increased together” when the copy number of the other gene (*CDK6* or *BRAF*) ≥ 2.2, and the difference with *MET* amplification is within +/−0.5.
(c) *MET* amplification that satisfies both (a) and (b) is defined as focal.

Results of analyzing patient cohort consisting with the testing cohort (291 patients), and validation cohort (734 patients) showed *MET* alterations related to abnormal signaling in approximately 10.7% (110 patients) of the entire patient population across nine different cancer types, most notably non-small cell and small cell lung cancers, gastroesophageal cancer, and prostate adenocarcinoma. *MET* alterations were found in 37 out of 291 patients. Among these, 24 had amplifications, 5 had exon 14 skipping, and 13 had single nucleotide variants (SNVs). Co-alterations, such as amplification, SNVs, were found in four samples.

Of the 24 *MET* amplifications, about 30% (7/24) were classified as focal amplification. The *MET* copy number was significantly higher with focal amplification compared to non-focal amplification (polysomy). In a validation cohort, focal *MET* amplification was detected in 4.2% of patients. Overall, the rate of focal amplification was 3.7% (=38/1025) across all patients.

This study showed that this approach can distinguish focal from non-focal *MET* amplification using comprehensive genomic profiling with NGS in patients with advanced cancer. The study also suggests that only 30% of all *MET* amplifications detected by the NGS are focal, and these are associated with a higher plasma *MET* copy number.

Nevertheless, it is pertinent to acknowledge a limitation inherent in this study, namely the selective focus on a restricted gene set localized within chromosome 7, encompassing *MET*, *EGFR*, *BRAF*, and *CDK6*. Inclusion of a broader array of genes or single nucleotide polymorphisms (SNPs) has the potential to substantially enhance the precision and fidelity of detecting focal amplification events. Furthermore, the absence of a well-defined correlation between *EGFR* and *MET* necessitates a more comprehensive exposition.

Although clinical investigations are warranted, NGS holds promise as a valuable tool for *MET* amplification analysis due to its widespread use in clinical practice and the potential to simultaneously examine other driver genes, such as *EGFR*. The integration of NGS with established methods such as FISH has been proposed in HER2-positive cases to capture those missed by the current IHC and FISH-based approaches [69]. By leveraging the strengths of both techniques, NGS-based detection may enhance the sensitivity and accuracy of *MET* amplification identification, leading to improved patient stratification and personalized treatment decisions.

To fully exploit the potential of NGS in *MET* amplification assessment, further research is needed to establish standardized guidelines for interpreting NGS results, particularly in distinguishing between polysomy and focal amplification. Additionally, the development of bioinformatics algorithms and computational tools specific to *MET* amplification analysis will aid in the accurate classification of NGS data. Efforts to validate the clinical utility of NGS-based detection of focal amplification and its correlation with treatment response are crucial steps toward integrating NGS into routine clinical practice for precision cancer care.

## 6. *MET* Amplification as an Acquired Resistance Mechanism

*MET* amplification has emerged as a significant mechanism of acquired resistance in various targeted therapies, particularly in *EGFR*-mutant NSCLCs. *MET* amplification is known to stimulate signaling pathways such as RAS-ERK/MAPK, STAT, and PI3K/AKT downstream of EGFR, leading to resistance against EGFR TKIs [70,71]. This resistance mechanism has been observed across all generations of EGFR TKIs [72,73,74].

Clinical studies have shed light on the prevalence of *MET* amplification as a resistance mechanism in NSCLC. Coleman et al. conducted a comprehensive analysis of key clinical trials in NSCLC and reported that *MET* amplification was identified as a mechanism of resistance in 7–15% of patients who experienced treatment failure with first-line osimertinib and in 10–22% following second-line osimertinib [26]. These findings highlight the clinical relevance of *MET* amplification in acquired resistance scenarios.

Moreover, it has been recognized that *MET* amplification may contribute to treatment resistance not only in *EGFR*-positive NSCLCs but also in other oncogenic gene-positive NSCLCs, such as those with *ALK* fusion [75], *RET*-fusion [76,77], *ROS1*-fusion [78], *NTRK*-fusion [79], or *KRAS* [80]. Collectively, it is estimated that approximately 15% of NSCLC tumors harboring *EGFR*, *KRAS*, *ALK* fusion, and *RET* fusion alterations exhibit *MET* amplification [26]. This underscores the importance of considering *MET* amplification as a potential resistance mechanism in the management of various oncogenic driver alterations.

Understanding the prevalence and clinical implications of *MET* amplification in acquired resistance scenarios is crucial for optimizing treatment strategies. Efforts are underway to develop effective therapeutic approaches targeting *MET* amplification to overcome acquired resistance. These include the investigation of combination therapies involving MET inhibitors with other targeted agents or immunotherapies to enhance treatment efficacy and prevent the emergence of resistance. Additionally, ongoing research aims to elucidate the underlying mechanisms and molecular interactions associated with *MET* amplification-mediated resistance, which will inform the development of novel therapeutic strategies for patients with *MET*-amplified NSCLC.

## 7. Treatment Option Targeting for MET Amplification

### 7.1. Monotherapy

As previously discussed, initial studies have indicated limited effectiveness of MET inhibitors in patients with *MET* amplification (*MET*/CEP ≥ 2.0) [62]. However, subsequent research has demonstrated that the efficacy of MET inhibitors improves with higher levels of amplification [18,81,82,83,84,85,86,87,88].

Table 1 summarizes the clinical trials investigating monotherapy targeting *MET* amplification. For instance, the PROFILE 1001 study examined the activity of crizotinib, an ALK/ROS1/MET tyrosine kinase inhibitor (TKI), in NSCLC patients categorized into high (*MET*/CEP ≥ 4), medium (4 > *MET*/CEP > 2.2), or low (2.2 ≥ *MET*/CEP ≥ 1.8) amplification groups [84]. The high-amplification group exhibited the highest objective response rate (ORR) of 38.1% and median progression-free survival (mPFS) of 6.7 months.

Another study evaluated the impact of capmatinib in NSCLC patients with *MET* amplification, revealing ORR rates of 29% (GCN ≥ 10), 12% (GCN 6 to 9), 9% (GCN 4 or 5), and 7% (GCN < 4), indicating an increased therapeutic effect with higher degrees of *MET* amplification (GEOMETRY mono-1 study, phase II) [18]. Several other MET-targeted TKIs have also been assessed in relation to the degree of *MET* amplification, consistently showing improved outcomes in the higher amplification groups (Table 1).

However, it is worth noting that the cutoff criteria for defining amplification varied across trials, and establishing standardized criteria for identifying optimal treatment targets remains a future challenge.

### 7.2. Combination Therapy

Combination therapy involving primary oncogene TKIs and MET TKIs has emerged as a rational strategy for addressing acquired resistance resulting from *MET* amplification. In recent years, significant advancements have been made in targeting both MET and EGFR, leading to the initiation of several clinical trials investigating the combination of MET inhibitors and EGFR inhibitors for acquired resistance in NSCLC. Table 2 provides an overview of combination therapy trials targeting *MET* amplification in *EGFR*-mutant NSCLC.

Capmatinib and tepotinib, both FDA-approved for NSCLC with *MET* exon14 skipping [18,19], have demonstrated positive outcomes when combined with gefitinib in pretreated NSCLC cases harboring both *EGFR* mutations and *MET* amplification [89,90]. In a phase II study, the combination of capmatinib and gefitinib in patients with acquired resistance to EGFR-TKI and *MET* amplification (GCN ≥ 4 by FISH) or MET overexpression (IHC 3+) showed improved activity, particularly in the high *MET* amplification group (GCN ≥ 6; ORR 47% and mPFS 5.5 months) [89]. Another phase II study, known as the INSIGHT study, investigated the combination of tepotinib and gefitinib in patients with acquired resistance to EGFR-TKI, *MET* amplification (GCN ≥ 5 or *MET*/CEP ≥ 2.0 by FISH), or MET overexpression (IHC 2+/3+). The ORR in this study was 67%, with a median PFS of 16.6 months compared to 4.2 months with chemotherapy (HR = 0.13, 90% CI = 0.04–0.43). Additionally, the overall survival was 37.3 months vs. 13.1 months with chemotherapy (HR = 0.08, 90% CI = 0.01–0.51) [90]. 

Savolitinib, a potent and selective MET TKI, in combination with osimertinib, was evaluated in the TATTON study for its efficacy in pretreated *EGFR* mutation-positive lung cancers with *MET* amplification [91]. In patients who experienced resistance after first- or second-generation EGFR-TKIs with *MET* amplification (GCN ≥ 5 or *MET*/CEP ≥ 2.0 by FISH or GCN ≥ 5 by NGS), the ORR ranged from 64% to 67%. For patients with resistance after third-generation EGFR-TKIs and *MET* amplification, the objective response rate was 30%. A phase I study examining the combination of savolitinib and gefitinib in patients with acquired resistance to EGFR-TKI and *MET* amplification (GCN ≥ 5 or *MET*/CEP ≥ 2.0 by FISH) reported an objective response rate of 52% [92]. It is important to note that the criteria for defining *MET* amplification varied across these studies. Furthermore, resistance-related amplification is often considered a subclonal population, which may warrant a lower threshold for defining the degree of amplification compared to de novo amplification [57,58]. Nevertheless, studies have consistently demonstrated that higher degrees of amplification correspond to improved treatment efficacy [57].

Ongoing investigations are exploring the use of antibody-based therapies targeting MET. Telisotuzumab vedotin, an antibody-drug conjugate targeting MET, exhibited promising antitumor activity and acceptable toxicity in NSCLC cases with acquired resistance to EGFR-TKI and MET overexpression (H-score ≥ 150 by IHC). In a study involving patients with MET overexpression (including four patients with *MET* amplification out of a total of 28 patients), ORR was 32%, and mPFS was 5.9 months [50].

### 7.3. Ongoing Study

Several ongoing clinical trials are currently investigating treatment strategies targeting *MET* amplification. These trials aim to further evaluate the efficacy and safety of different therapeutic approaches. One notable trial is a phase III study evaluating the combination of savolitinib and osimertinib for acquired resistance in NSCLC (ClinicalTrials.gov Identifier: NCT05015608). This trial seeks to assess the potential benefits of combining a MET inhibitor (savolitinib) with a third-generation EGFR inhibitor (osimertinib) in patients who have developed resistance to previous treatments.

In addition, a phase II trial is underway to investigate the efficacy of savolitinib in combination with osimertinib for acquired resistance in NSCLC (ClinicalTrials.gov Identifier: NCT03778229). This study aims to provide further insights into the potential synergistic effects of these targeted therapies in overcoming resistance mechanisms, specifically in patients with *MET* amplification.

Furthermore, a phase II trial is evaluating the combination of savolitinib and durvalumab, an immune checkpoint inhibitor, for advanced gastric cancer (ClinicalTrials.gov Identifier: NCT05620628). This trial explores the potential of combining MET inhibition with immune checkpoint blockade to enhance therapeutic outcomes in this patient population.

Lastly, a phase I trial is investigating amivantamab, a human bispecific antibody targeting both EGFR and MET, in advanced NSCLC (ClinicalTrials.gov Identifier: NCT02609776). This study aims to assess the safety and efficacy of this novel antibody therapy in patients with advanced NSCLC harboring *EGFR* mutations and *MET* amplification.

These ongoing trials represent important steps in advancing our understanding of *MET* amplification as a therapeutic target and may contribute to the development of more effective treatment strategies for patients with acquired resistance in various cancer types. The results from these studies will provide valuable insights into the clinical utility of targeting *MET* amplification and may guide future treatment decisions in precision oncology.

## 8. Conclusions

The treatment of *MET* amplification represents a promising avenue, particularly in combination with EGFR-TKI therapy for pretreated NSCLC patients. However, the appropriate selection of patients is crucial for maximizing treatment efficacy. The underlying mechanisms contribute to the varying effectiveness of MET inhibitors based on the degree of *MET* amplification. Nevertheless, there remains a lack of consensus regarding the specific threshold or cut-off value for *MET* amplification across different studies.

Further research is needed to determine the optimal cutoff value for *MET* amplification, to identify the best combination therapies, and to develop new targeted therapies for patients with *MET* amplification.

## Figures and Tables

**Table 1:** *MET* amplification targeting therapy trials (monotherapy).

Drug	Cancer Type	Study	*MET* Amplification Criteria	Clinical Outcome	
SAR125844	Solid tumors	Phase I (n = 72) Angevin et al., 2017 [81]	GCN > 4 and *MET*/CEP ≥ 2.0 by FISH (or IHC 2+/3+)	ORR 17% (5/29)	
AMG337	Gastric cancers	Phase II (n = 60) Van Cutsem et al., 2019 [82]	*MET*/CEP ≥ 2.0 by FISH	ORR 18% (8/45)	
	Solid tumors	Phase I (n = 111) Hong et al., 2019 [83]	*MET*/CEP ≥ 2.0 by FISH	4 > *MET*/CEP	ORR 0% (0/2)
				*MET*/CEP ≥ 4	ORR 60% (6/10)
crizotinib	NSCLC	Phase I (n = 38) [PROFILE 1001] Camidge et al., 2021 [84]	*MET*/CEP ≥ 1.8 by FISH	2.2 ≥ *MET*/CEP ≥ 1.8	ORR 33% (1/3) mPFS 1.8 months
				4.0 > *MET*/CEP > 2.2	ORR 14% (2/14) mPFS 1.9 months
				*MET*/CEP ≥ 4.0	ORR 38% (8/21) mPFS 6.7 months
		Phase II (n = 26) [METROS] Landi et al., 2019 [85]	*MET*/CEP > 2.2 by FISH	5.0 > *MET*/CEP > 2.2	ORR 36% (5/14)
				*MET*/CEP ≥ 5.0	ORR 0% (0/2)
		Phase II (n = 25) [AcSe] Moro-Sibilot et al., 2019 [86]	GCN ≥ 6 by FISH	ORR 16% (4/25) mPFS 3.2 months	
capmatinib	NSCLC	Phase I (n = 44) Schuler et al., 2020 [87]	GCN ≥ 5 or *MET*/CEP ≥ 2.0 by FISH (or IHC 2+/3+ or H-score ≥ 150)	4 > GCN	ORR 0% (0/17)
				6 > GCN ≥ 4	ORR 17% (2/12)
				GCN ≥ 6	ORR 47% (7/15) mPFS 9.3 months
		Phase II (n = 195) [GEOMETRY mono-1] Wolf et al., 2020 [18]	determined by FISH, NGS	4 > GCN	ORR 7% (2/30) mPFS 3.6 months
				GCN 4 or 5	ORR 9% (5/54) mPFS 2.7 months
				GCN 6 to 9	ORR 12% (5/42) mPFS 2.7 months
				GCN ≥ 10	ORR 29% (20/69) mPFS 4.1 months
	Hepatocellular carcinoma	Phase II (n = 30) Qin et al., 2019 [88]	GCN ≥ 5 or *MET*/CEP ≥ 2.0 by FISH (or IHC 2+/3+)	ORR 10% (3/30)	

ORR; objective response rate, mPFS; median progression free survival.

**Table 2:** *MET* amplification combination therapy trials for *EGFR*-mutant NSCLC.

Drug	Study	Patient/*MET* Amplification Criteria	Clinical Outcome	
capmatinib/gefitinib	Phase II (n = 100) Wu et al., 2018 [89]	acquired resistance to EGFR-TKI GCN ≥ 4 by FISH (or IHC 3+)	4 > GCN	ORR 12% (5/41) mPFS 3.9 months
			6 > GCN ≥ 4	ORR 22% (4/18) mPFS 5.4 months
			GCN ≥ 6	ORR 47% (17/36) mPFS 5.5 months
tepotinib/gefitinib	Phase II (n = 12) [INSIGHIT] Wu et al., 2020 [90]	acquired resistance to EGFR-TKI and T790 negative GCN ≥ 5 or *MET*/CEP ≥ 2.0 by FISH (or IHC 2+/3+)	ORR 67% (8/12) mPFS 16.6mo (vs. 4.2 months with chemotherapy, HR = 0.13, 90% CI = 0.04–0.43) OS 37.3mo (vs. 13.1 months with chemotherapy, HR = 0.08, 90% CI = 0.01–0.51)	
savolitinib/osimertinib	Phase Ib (n = 174) [TATTON] Sequist et al., 2020 [91]	acquired resistance to EGFR-TKI GCN ≥ 5 or *MET*/CEP ≥ 2.0 by FISH or GCN ≥ 5 by NGS	Cohort B1 *; after 3rd gen. EGFR-TKI and T790 negative	ORR 30% (21/69) mPFS 5.4 months
			Cohort B2 *; after 1st/2nd gen. EGFR-TKI and T790 negative	ORR 65% (33/51) mPFS 9.0 months
			Cohort B3 *; after 1st/2nd gen. EGFR-TKI and T790 positive	ORR 67% (12/18) mPFS 11.0 months
			Cohort D **; after 1st/2nd gen. EGFR-TKI and T790 negative	ORR 64% (23/36) mPFS 9.1 months
savolitinib/gefitinib	Phase I (n = 57) Yang et al., 2021 [92]	acquired resistance to EGFR-TKI and T790 negative GCN ≥ 5 or *MET*/CEP ≥ 2.0 by FISH	ORR 52% (12/23)	
Telisotuzumab vedotin/erlotinib	Phase Ib (n = 28) *** Camidge et al., 2023 [50]	acquired resistance to EGFR-TKI (H-score ≥ 150 by IHC)	ORR 32% (9/28) mPFS 5.9 months	

* savolitinib 600/300 mg plus osimertinib 80 mg. ** savolitinib 300 mg plus osimertinib 80 mg. *** including 4 *MET* amplified. ORR; objective response rate, mPFS; median progression free survival, OS; overall survival.

## Data Availability

The data presented in this study are available in this article.
